# Non-Coding RNAs in Gastrointestinal Stromal Tumors: Regulatory Networks, Drug Resistance, and Clinical Implications

**DOI:** 10.3390/ijms27167286

**Published:** 2026-08-15

**Authors:** Georgios Mandrakis, Stavros P. Papadakos, Georgia Levidou, Panoraia Keratsa, Maria-Ioanna Christodoulou, Stamatios Theocharis

**Affiliations:** 1 First Department of Pathology, School of Medicine, National and Kapodistrian University of Athens, 10679 Athens, Greece; stpap@med.uoa.gr (S.P.P.); panorke@med.uoa.gr (P.K.); 2 Department of Pathology, Paracelsus Medical University, 90419 Nuremberg, Germany; georgia.levidou@klinikum-nuernberg.de; 3 Tumor Immunology and Biomarkers Laboratory, Basic and Translational Cancer Research Center, Department of Life Sciences, European University Cyprus, Nicosia 2404, Cyprus; mar.christodoulou@euc.ac.cy

**Keywords:** gastrointestinal stromal tumors, ncRNAs, miRNAs, lncRNAs, circRNAs, TKI resistance, KIT, PDGFRA, precision oncology

## Abstract

Gastrointestinal stromal tumors (GISTs) are the most common mesenchymal tumors of the gastrointestinal tract and are usually driven by activating mutations in KIT or PDGFRA. Although these alterations define the core molecular biology of GISTs and guide targeted therapy, they do not fully explain the variability observed in tumor behavior, recurrence risk, or response to tyrosine kinase inhibitors. Non-coding RNAs (ncRNAs), including microRNAs (miRNAs), long non-coding RNAs (lncRNAs), and circular RNAs (circRNAs), have been increasingly studied as regulators of gene expression in GISTs. Recent evidence suggests that these molecules may influence KIT-centered signaling, autophagy, apoptosis, invasion, angiogenesis, and drug resistance. However, the strength of evidence differs considerably across individual ncRNAs, ranging from bioinformatic associations to functional validation in cell lines and in vivo models. This review summarizes current knowledge on ncRNA-mediated regulation in GIST biology, with emphasis on tumor progression, therapeutic resistance, and possible clinical relevance. Rather than treating ncRNAs as isolated biomarkers, they function as part of broader regulatory networks that interact with oncogenic signaling and epigenetic mechanisms. Although several ncRNAs appear promising as prognostic or predictive candidates, further validation in independent clinical cohorts is required before their integration into routine risk stratification or treatment decision-making.

## 1. Introduction

Gastrointestinal stromal tumors (GISTs) represent the major mesenchymal tumor type of the gastrointestinal tract, although they account for only a small fraction of all gastrointestinal malignancies [1]. They are thought to arise from, or share a common precursor with, the interstitial cells of Cajal, which are involved in the regulation of gut motility [2]. Their reported annual incidence varies between studies, generally ranging from approximately 0.78 to 2 cases per 100,000 individuals, and they are most frequently diagnosed in older adults [3,4].

Anatomically, GISTs occur most often in the stomach, followed by the small intestine, whereas tumors of the colon, rectum, esophagus, and omentum are less common [3,4]. The 5-year overall survival (OS) rate depends on tumor location in the gastrointestinal tract, particularly in the stomach, jejunum, and ileum, as well as on treatment strategies such as surgery and systemic therapy [5]. Their clinical presentation is variable and depends largely on tumor size and location. Some patients are asymptomatic, whereas others present with gastrointestinal bleeding, anemia, dyspepsia, abdominal pain, or obstructive symptoms [3]. Histologically, GISTs are usually classified into spindle-cell, epithelioid, or mixed subtypes, with spindle-cell morphology being the most frequent [3].

Despite major advances in understanding the genetic drivers of GISTs, the regulatory mechanisms that contribute to tumor heterogeneity, progression, and therapeutic resistance remain incompletely understood. In this context, non-coding RNAs (ncRNAs) have emerged as important regulators of gene expression and tumor biology. Against this background, a recent review by Ma et al. focused on ncRNA-mediated regulation of programmed cell-death pathways, particularly apoptosis, autophagy, and ferroptosis, and their relationship to drug resistance in GISTs [6]. The present review adopts a complementary, broader framework by integrating miRNAs, lncRNAs, and circRNAs across KIT/PDGFRA-centered signaling, tumor progression, invasion and metastasis, angiogenesis, epigenetic regulation, and therapeutic resistance. Beyond providing dedicated coverage of circRNA networks and integrating the epigenetic, transcriptional, and post-transcriptional mechanisms that regulate ncRNA expression, it explicitly distinguishes predominantly computational associations from findings supported by functional experiments. Furthermore, it extends the analysis toward clinical translation by proposing a hypothesis-generating multiplex ncRNA panel for future validation in risk stratification and prediction of TKI response.

## 2. Molecular and Genetic Landscape of GISTs

GISTs are mainly characterized by gain-of-function mutations in receptor tyrosine kinases, most commonly KIT, which occurs in about 75–80% of cases, or platelet-derived growth factor receptor-alpha (PDGFRA), which occurs in about 5–10% of cases. These mutations lead to constitutive activation of key downstream signaling pathways such as MAPK, PI3K/AKT/mTOR, and STAT3, thereby promoting tumor cell proliferation, survival, and oncogenic transformation [7,8,9]. In addition to these oncogenic drivers, the transcription factor ETV1 plays a critical role in the growth and survival of GIST cells, acting as a lineage-specific survival factor that cooperates with KIT signaling to maintain the GIST phenotype [10].

Immunohistochemistry remains central to GIST diagnosis. Most tumors show KIT positivity, while immunohistochemical markers such as DOG1 and CD34 serve as important complementary diagnostic markers, particularly in tumors lacking detectable KIT expression [3]. These mutations are considered the primary drivers of GIST pathogenesis, determining whether the tumors are resistant or sensitive to tyrosine kinase inhibitor (TKI) therapy such as imatinib mesylate, and such cases are commonly referred to as mutant GISTs [11]. However, a subset of GISTs lacks canonical KIT or PDGFRA mutations and displays alternative oncogenic mechanisms.

Around 10% to 15% of adult GISTs, however, do not harbor mutations in these genes, classifying them as wild-type GISTs [11]. This group is molecularly heterogeneous and includes several biologically distinct subtypes. One important category is succinate dehydrogenase (SDH)-deficient GISTs, which are characterized by loss of function of the mitochondrial SDH complex, often caused by mutations in SDHA, SDHB, SDHC, or SDHD. SDH-deficient GISTs frequently occur in younger patients and may be associated with hereditary Carney–Stratakis syndrome or the non-hereditary Carney triad. These tumors display distinct molecular and clinical characteristics, including activation of hypoxia-related pathways, widespread epigenetic alterations, and relative resistance to conventional TKI therapy [12,13].

Within the wild-type group, a smaller subset known as quadruple wild-type GISTs lacks detectable alterations in KIT, PDGFRA, SDH, or RAS pathway genes, further highlighting the molecular heterogeneity of these tumors [14]. Additional rare oncogenic drivers have also been described in wild-type GISTs. Mutations in components of the RAS/RAF signaling pathway, including BRAF, have been reported in a small proportion of cases [15,16]. Furthermore, gene fusions involving neurotrophic tyrosine receptor kinase (NTRK) genes have been identified in rare GISTs and represent potentially actionable therapeutic targets [17].

Localized wild-type GISTs are primarily managed surgically, whereas systemic treatment of advanced disease should be guided by the underlying molecular subtype rather than applied uniformly with imatinib [18,19]. Although immunotherapy is not yet part of standard GIST management, several trials are exploring its potential benefits [20]. Ongoing research remains essential, as current therapeutic strategies, while effective for many patients, still face challenges due to heterogeneous treatment responses and drug resistance [21].

During TKI treatment, secondary mutations in KIT may arise, particularly in the kinase domains encoded by exons 13, 14, 17 and 18, contributing to acquired resistance and disease progression. More specifically, secondary KIT mutations commonly affect the ATP-binding pocket encoded by exons 13 and 14 or the activation loop encoded by exons 17 and 18 [22]. These secondary alterations can modify kinase conformation and reduce the inhibitory activity of first-line therapies, representing a major challenge in long-term disease management [23,24,25].

Established GIST recurrence-risk models incorporate tumor size, mitotic rate, anatomical site, and tumor rupture [19]. Ki-67 may provide additional prognostic information, although it has not replaced these established criteria [26].

Although KIT and PDGFRA are key proteins for the development of GIST, other proteins have been implicated in influencing both metastatic potential and response to imatinib therapy. For example, downregulation of insulin-like growth factor-1 receptor (IGF-1R) led to reduced cell viability in GIST cells after imatinib treatment [27]. Moreover, IGF-1R is overexpressed in wild-type and pediatric GISTs, revealing an oncogenic role in patients with wildtype GIST [28].

Because aberrant KIT and PDGFRA signaling represents the principal oncogenic driver in most GISTs, pharmacological inhibition of these kinases has become the cornerstone of systemic therapy. The development of tyrosine kinase inhibitors has therefore profoundly changed the therapeutic landscape of GISTs, although treatment efficacy is frequently limited by primary and acquired resistance mechanisms. These therapeutic strategies and the molecular basis of resistance are discussed in the following section.

## 3. TKI Therapy and Resistance in GISTs

Historically, GISTs have been challenging to treat with conventional chemotherapy due to their inherent resistance to standard cytotoxic drugs [21]. However, GIST treatment was revolutionized by the introduction of TKIs, notably imatinib, which specifically targets oncogenic mutations in KIT and PDGFRA receptors [21]. This approach remains central to GIST management, as accurate molecular characterization is needed to guide treatment selection.

Imatinib is effective against most KIT and PDGFRA mutations, with KIT exon 11 mutations showing the highest sensitivity to treatment [21]. In advanced or metastatic GIST, acquired imatinib resistance commonly emerges after approximately 18–24 months and the standard subsequent sequence is sunitinib, regorafenib, and ripretinib, whereas avapritinib is preferred for PDGFRA D842V-mutant disease [19,29].

In addition to oncogenic signaling, angiogenesis contributes to GIST progression. It is driven mainly by vascular endothelial growth factor (VEGF) and PDGF signaling, which mediate tumor-associated neovascularization. The angiogenic process is orchestrated by the balance of pro- and anti-angiogenic factors in the tumor microenvironment (TME), driven largely by hypoxia-inducible factor-1α (HIF-1α) and the VEGF/VEGFR axis [3]. FGF2-driven activation of VEGF signaling may contribute to imatinib resistance in GISTs [30].

While secondary KIT or PDGFRA mutations represent the most common cause of resistance, accumulating evidence suggests that epigenetic mechanisms may also contribute. Resistance to sunitinib, a second-line TKI for imatinib-refractory GISTs, has been linked to PTEN promoter hypermethylation, which silences PTEN and sustains AKT/mTOR signaling despite KIT inhibition [31]. Restoring PTEN expression partially resensitized resistant cells, while knockdown reproduced the resistant phenotype, underscoring PTEN’s role in drug response [31]. The partial restoration of drug sensitivity after PTEN re-expression suggests that this mechanism is relevant, although it is unlikely to explain resistance on its own.

## 4. Tumor Microenvironment and Immunotherapy in GISTs

TME in GISTs represents a complex network of immune interactions, characterized by infiltration of cytotoxic CD8^+^ T-cells, regulatory T-cells (Tregs), and tumor-associated macrophages (TAMs) [20]. This immune balance contributes to both tumor progression and immunosuppression, indoleamine 2,3-dioxygenase (IDO) overexpression, major histocompatibility complex class I (MHC-I) downregulation, and Treg activation [20]. Interestingly, imatinib also exerts immunomodulatory effects by enhancing NK-cell and CD8^+^ T-cell activity, while reducing the presence of Tregs, and decreasing IDO expression, collectively promoting anti-tumor immune responses [20]. These findings support ongoing research into combining TKIs with immunotherapeutic approaches, including immune checkpoint inhibitors (ICIs) and cytokine-based immunotherapies, which are currently being investigated in clinical trials [4,20].

Despite major advances in understanding the genetic drivers of GISTs and the development of targeted therapies, several aspects of GIST biology remain incompletely understood. Tumor heterogeneity, variable therapeutic responses, and mechanisms of drug resistance are influenced not only by genomic alterations but also by additional regulatory layers that modulate gene expression and cellular signaling. In recent years, ncRNAs have emerged as key regulators of these processes, affecting oncogenic signaling pathways, tumor–microenvironment interactions, and therapeutic response.

## 5. Can Non-Coding RNAs Bridge the Knowledge Gap in GIST Biology?

Despite substantial advances in the diagnosis and treatment of GISTs, critical gaps remain in our understanding of their underlying molecular biology, particularly regarding mechanisms of drug resistance, angiogenic adaptation, and immune evasion. While integrated molecular, immunological, and imaging approaches have yielded important insights, additional in vivo studies and functional investigations using GIST-derived cell lines are required to clarify how these processes interact within the TME [32].

In this context, ncRNAs have emerged as key regulators capable of modulating oncogenic signaling, angiogenesis, and tumor behavior at both transcriptional and post-transcriptional levels [33]. Increasing evidence supports their role as integral components of the genetic and epigenetic regulatory landscape of GISTs, with potential diagnostic and prognostic relevance [34].

At a broad functional level, ncRNAs are traditionally classified into housekeeping ncRNAs, including rRNAs, tRNAs, snRNAs, and snoRNAs, which support essential cellular processes, and regulatory ncRNAs [35]. The latter include miRNAs, siRNAs, piRNAs, lncRNAs, and circRNAs. Specifically, the present review focuses primarily on miRNAs, lncRNAs, and circRNAs because these represent the best-characterized regulatory ncRNA classes in GIST.

Compared with protein-based biomarkers, such as annexin V, HMGB1, C13orf2, glutamate dehydrogenase 1, fibrinogen beta chain, and RoXaN, which show altered expression across GIST grades [36], RNA-based markers offer several technical and biological advantages. Antibody-based protein detection methods often suffer from limited specificity and sensitivity due to variability in antibody performance and non-specific binding, which can complicate accurate assessment of tumor heterogeneity [37].

In contrast, RNA profiling enables sensitive and quantitative characterization of tumor-specific molecular states using targeted RT-qPCR and bulk RNA sequencing [38]. State-of-the-art approaches, including single-cell RNA sequencing and spatial transcriptomics, can further resolve cell-type-specific expression programs and preserve the spatial organization of tumor–microenvironment interactions [39]. In GIST, single-cell RNA sequencing has already revealed transcriptional heterogeneity and distinct immune microenvironments associated with imatinib resistance [40]. Although the capture efficiency of individual ncRNA classes depends on the platform and library-preparation strategy, integrating these technologies with ncRNA-focused assays may refine tumor classification and prognostic stratification and facilitate clinical translation.

## 6. Functional Roles of Non-Coding RNAs in GIST Biology

Beyond protein-coding genes, the human genome encodes a diverse repertoire of ncRNAs that play fundamental roles in gene regulation and disease pathogenesis, including cancer [41]. Major ncRNA classes include microRNAs (miRNAs), long non-coding RNAs (lncRNAs), and circular RNAs (circRNAs), which are traditionally classified as non-coding and regulate gene expression through diverse mechanisms. For consistency, miRNA nomenclature was harmonized according to the original studies and cross-checked against miRBase, with mature strand notation (-5p/-3p) used where supported [42].

miRNAs are short (~20–25 nt) RNAs that post-transcriptionally regulate gene expression by binding to target mRNAs, leading to translational repression or mRNA degradation [43,44,45]. Their biogenesis involves sequential processing by the Drosha/DGCR8 and Dicer, ultimately generating mature miRNAs capable of targeting multiple mRNAs, thereby contributing to regulatory complexity and tumor heterogeneity [46,47].

**Figure 1:**
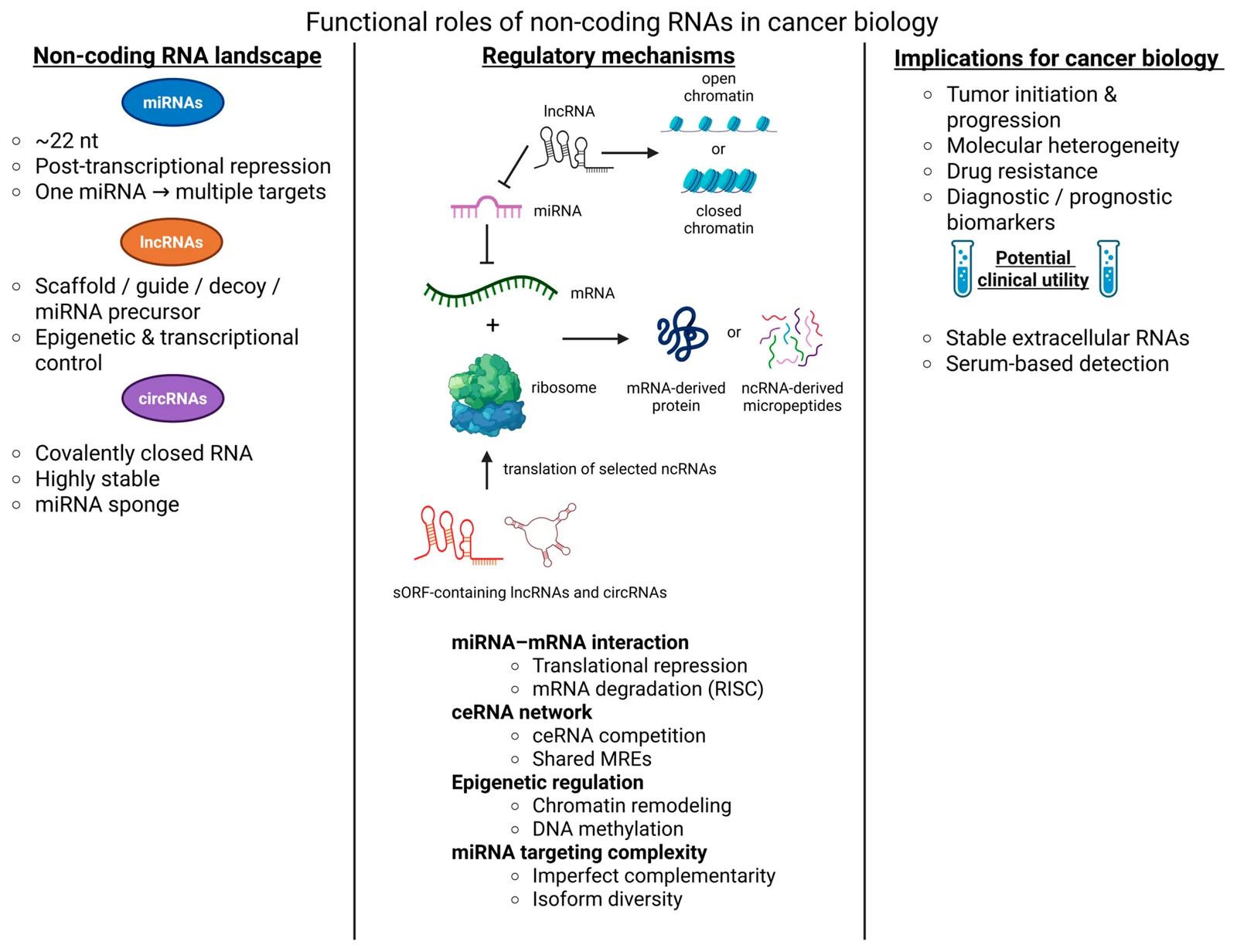
Overview of major ncRNA classes and their regulatory roles in cancer biology. miRNAs, lncRNAs, and circRNAs regulate gene expression through post-transcriptional repression, chromatin and epigenetic regulation, and ceRNA interactions. In addition, selected sORF-containing lncRNAs and circRNAs can associate with ribosomes and generate functional micropeptides. These mechanisms influence tumor initiation and progression, molecular heterogeneity, drug resistance, and biomarker potential. Arrows indicate the direction of the depicted regulatory or biological processes.Created in BioRender. Mandrakis, G. (2026) https://BioRender.com/wq9ruea (accessed on 12 August 2026).

LncRNAs are longer transcripts (>200 nt) that regulate gene expression through diverse mechanisms, including scaffolding, guiding, and decoy functions, as well as epigenetic and chromatin-level regulatory mechanisms [41,48]. A key conceptual framework linking miRNAs and lncRNAs is the competing endogenous RNA (ceRNA) hypothesis, whereby mRNAs, lncRNAs, and pseudogene transcripts communicate through shared miRNA response elements (MREs), competing for miRNA binding and thereby modulating protein output [49,50,51]. These ceRNA networks add an additional post-transcriptional regulatory layer that can promote either oncogenic or tumor-suppressive programs, depending on cellular context (Figure 1) [52].

CircRNAs are stable ncRNAs generated by back-splicing, forming covalently closed molecules with regulatory functions in gene expression [53]. They can act as miRNA sponges, interact with RNA-binding proteins, and influence tumor-related pathways [54,55].

Recent discoveries have further expanded the ncRNA landscape by demonstrating that transcripts annotated as non-coding, particularly lncRNAs and circRNAs containing short open reading frames (sORFs), can produce functional micropeptides, often fewer than 100 amino acids [35,56]. Notably, two lncRNAs already implicated in GIST have demonstrated coding potential in other malignancies. H19, which is upregulated in GIST and correlates with ETV1 [57], encodes the 97-amino-acid micropeptide altH19 in multiple myeloma. Specifically, altH19 promotes DNA replication and mitotic progression by enhancing CDK2 phosphorylation at threonine 160 [58]. PVT1, which promotes GIST progression through PI3K/AKT/mTOR signaling [59], also gives rise in colorectal cancer to an HLA class I-restricted peptide recognized by tumor-reactive CD8+ T cells, linking lncRNA translation to antitumor immune surveillance [60]. Conversely, circPPP1R12A encodes a 73-amino-acid peptide, circPPP1R12A-73aa, that activates Hippo–YAP signaling and promotes colon cancer growth and metastasis [61]. To the best of our knowledge, no ncRNA-encoded micropeptide has yet been functionally characterized in GIST. Future studies integrating ribosome profiling and proteogenomic approaches may help clarify whether ncRNA-derived micropeptides also contribute to GIST biology.

ncRNA expression and activity are controlled at multiple levels, including epigenetic, transcriptional, and post-transcriptional regulation. In GIST, promoter methylation contributes to the silencing of tumor-suppressive miRNAs, CTCF activates transcription of the oncogenic lncRNA SNHG16, and RNA modifications influence ncRNA maturation and stability through METTL3-dependent m6A modification facilitating miR-25-3p maturation and FTO-mediated stabilization of XIST [62,63,64,65]. These mechanisms converge with ncRNA-mediated signaling networks that influence tumor progression and therapeutic response. The stability and detectability of miRNAs in biofluids support their potential for biofluid-based biomarker development [38,66].

Collectively, understanding ncRNA-mediated regulatory mechanisms in GISTs provides critical insights into tumor biology and highlights novel opportunities for biomarker development and therapeutic intervention. Among the different ncRNA classes, miRNAs represent the most extensively studied regulators of gene expression in GISTs and have been implicated in multiple aspects of tumor biology, including proliferation, invasion, and therapeutic response.

## 7. miRNAs Acting as Tumor Suppressors and Oncogenes in GISTs

### 7.1. miRNAs Targeting KIT and Oncogenic Signaling

MiRNAs in GISTs can function either as tumor suppressors or oncogenes depending on their target genes and the signaling pathways they regulate. Recent studies have identified multiple miRNAs as tumor suppressors in GISTs, implicating them in the regulation of proliferation, invasion, apoptosis, autophagy, angiogenesis, and response to imatinib (Table 1).

**Table 1:** Tumor-suppressive microRNAs involved in GIST biology and therapeutic response. For some miRNAs, molecular targets have not yet been fully characterized in GISTs and are therefore listed based on expression or functional studies. * Putative target/pathway based on currently available evidence; direct mechanistic validation in GIST models remains limited. ↑ indicates an increase, whereas ↓ indicates a decrease in the indicated biological effect.

miRNA	Functional Role	Molecular Target(s)/Pathway	Biological Effect	Reference
miR-375-3p	Tumor suppressor	KIT	↓ proliferation, migration	[67]
miR-200b-3p	Tumor suppressor	ETV1/EGFR signaling	↓ EGFR/ETV1 signaling, migration	[67]
miR-221-3p/miR-222-3p	Tumor suppressor	KIT, AKT, BCL2	↓ proliferation, ↑ apoptosis	[68]
miR-494-3p	Tumor suppressor	KIT	↓ KIT expression/signaling, ↓ proliferation	[69]
miR-125a-5p	Context-dependent	PTPN18	Imatinib resistance modulation	[70]
miR-128-3p	Tumor suppressor	CASC3	↓ proliferation, invasion	[64]
miR-148b-3p	Tumor suppressor	KIT/ERK/AKT/STAT3	↓ proliferation, ↓ signaling	[71]
miR-30a-5p	Tumor suppressor	BECLIN-1	↑ imatinib sensitivity	[72]
miR-130a-3p	Tumor suppressor	ATG2B	↑ imatinib sensitivity	[73]
miR-518a-5p	Tumor suppressor	PIK3C2A	↓ proliferation, ↑ apoptosis; loss associated with imatinib resistance	[74]
miR-4510	Tumor suppressor	APOC2	↓ invasion, ↓ AKT/ERK signaling	[75]
miR-218	Tumor suppressor	KIT	↑ apoptosis	[76]
miR-34a	Tumor suppressor	PDGFRA	↓ proliferation, migration, invasion	[62]
miR-335 *	Tumor suppressor	Not established	↓ proliferation	[62]
miR-144-3p *	Tumor suppressor	Predicted KIT/PLAT/ETV1 network	Predicted involvement in GIST regulatory networks	[77]
let-7e-5p	Prognostic miRNA	ECM/differentiation genes	Associated with relapse	[78]
miR-137-3p	Tumor suppressor	Twist1	↓ EMT, ↓ proliferation	[79]
miR-186-5p	Tumor suppressor	Not established; candidate are MET, AKT2, CXCR4, EFEMP1, and IGFBP3	↑ migration; ↑ recurrence risk; ↓ survival (when downregulated)	[80]
miR-6838-5p	Tumor suppressor	CDV3	Loss promotes proliferation and the Warburg effect	[81]
miR-520a-3p	Tumor suppressor	KIT within the XIST/miR-520a-3p/KIT axis	↓ proliferation, restores imatinib sensitivity	[65]
miR-409-5p	Tumor suppressor	KDM4D/HIF-1β/VEGF-A	↓ angiogenesis; enhances the antiangiogenic effect of imatinib	[82]

For example, miR-375-3p and miR-200b-3p are downregulated in GIST cells and display tumor-suppressive activity. miR-375-3p directly targets KIT and reduces cell viability and migration, whereas miR-200b-3p targets EGFR, decreases ETV1 protein expression, and suppresses cell migration [67].

Similarly, miR-221-3p and miR-222-3p are also significantly reduced in GISTs, particularly in wild-type tumors, and their restoration reduces KIT expression and phosphorylation, suppresses AKT signaling, reduces BCL2 levels, and induces apoptosis [68]. Moreover, miR-494-3p has also been reported to downregulate KIT and inhibit GIST cell proliferation, further supporting the role of KIT-targeting miRNAs in GIST biology [69].

Furthermore, bioinformatic analyses have identified miR-28-5p and miR-125a-5p as potential regulators of imatinib response, with increased expression associated with imatinib resistance [83]. However, functional validation is limited for miR-28-5p, and miR-125a-5p has been functionally linked to imatinib resistance in KIT-mutant GIST models through repression of PTPN18, supporting a context-dependent role [70].

**Figure 2:**
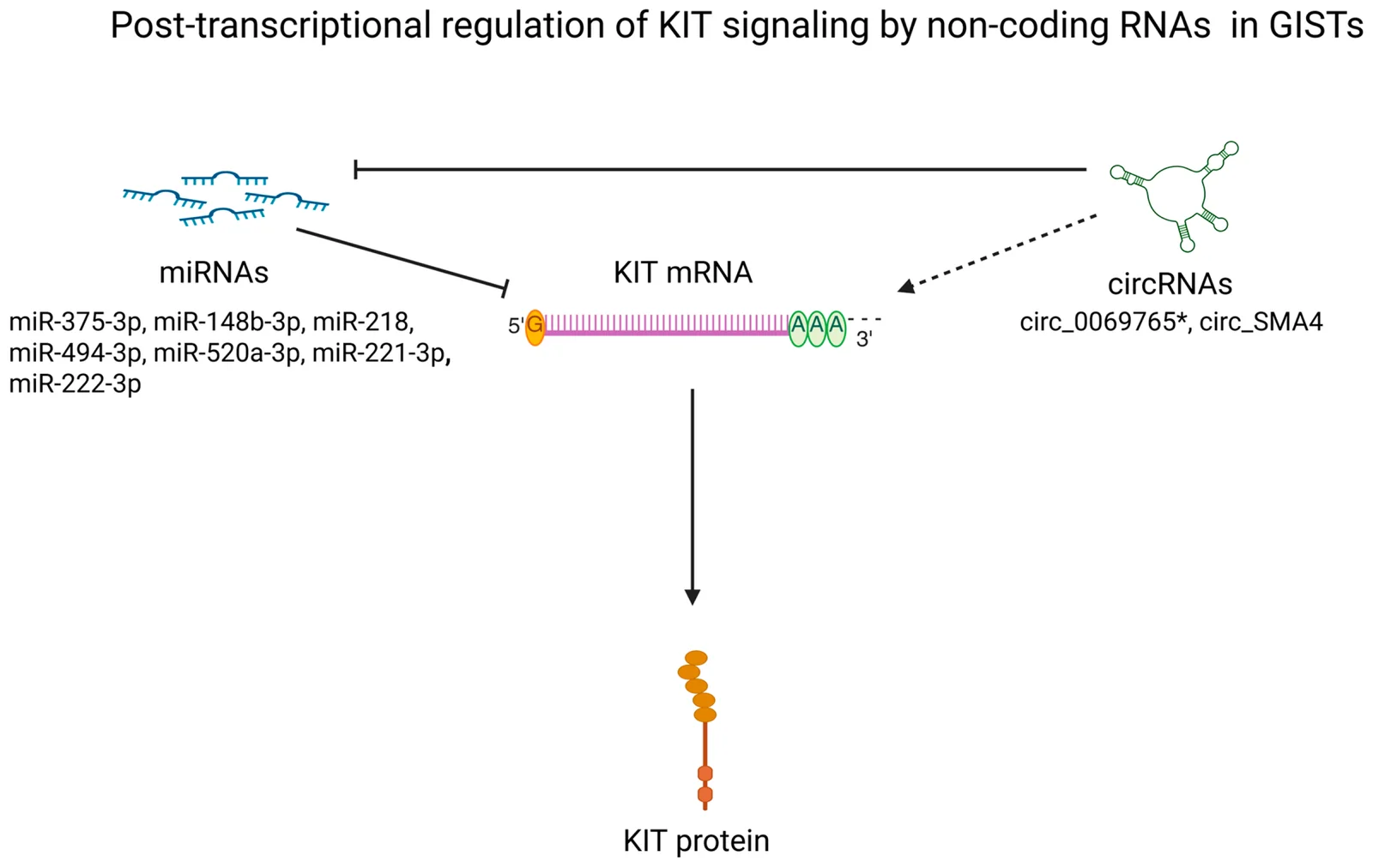
Post-transcriptional regulation of KIT signaling by ncRNAs in GISTs. Tumor-suppressive miRNAs (miR-375-3p, miR-148b-3p, miR-218, miR-494-3p, miR-520a-3p, miR-221-3p and miR-222-3p) directly target KIT mRNA, leading to reduced KIT protein expression and suppression of oncogenic signaling. In contrast, circRNAs such as circ_0069765 and circ_SMA4 function as ceRNAs, sequestering KIT-targeting miRNAs and thereby preserving KIT expression. Through these mechanisms, ncRNA networks fine-tune the activity of the KIT signaling axis in GISTs. Putative regulatory interactions based on computational evidence are denoted with an asterisk. Created in BioRender. Mandrakis, G. (2026) https://BioRender.com/mcwu4pl (accessed on 12 August 2026).

miR-128-3p is downregulated in GIST cell lines and functions as a tumor-suppressive miRNA. Its activity is antagonized by the oncogenic lncRNA SNHG16, which acts as a ceRNA and thereby derepresses the direct miR-128-3p target CASC3. Restoration of miR-128-3p inhibits proliferation, migration, and invasion and promotes apoptosis in GIST882 and GIST48 cells. Xenograft rescue experiments further support the oncogenic role of the SNHG16/CASC3 axis, although miR-128-3p itself was not directly manipulated in vivo [64]. Similarly, miR-148b-3p targets KIT and suppresses downstream signaling pathways, with reduced expression correlating with poorer clinical outcomes [71]. Collectively, these findings highlight the central role of miRNAs in regulating KIT signaling in GISTs, as summarized in Figure 2.

### 7.2. miRNAs Regulating Autophagy and Imatinib Response

Autophagy-related ncRNA mechanisms also contribute to tumor suppression in GISTs. For example, miR-30a-5p enhances imatinib sensitivity by targeting BECLIN-1 and suppressing autophagy [72]. In contrast, the lncRNA HOTAIR promotes autophagy-mediated survival by sponging miR-130a-3p and enabling ATG2B expression [73]. Moreover, miR-518a-5p was downregulated in matched imatinib-resistant GIST specimens, whereas restoration of expression in GIST882R cells directly represses PIK3C2A, reduces proliferation, and promotes apoptosis, supporting a tumor-suppressive role and implicating its loss in acquired imatinib resistance [74]. These mechanisms are discussed further in the context of drug resistance in Section 10.

### 7.3. miRNAs Regulating Apoptosis, EMT, and Tumor Progression

Additional tumor-suppressive miRNAs include miR-4510, which targets APOC2 and inhibits AKT/ERK signaling and invasion [75], and miR-218, which represses KIT and promotes apoptosis [76]. miR-34a and miR-335 were identified as epigenetically silenced candidate tumor suppressors, with restoration of either miRNA suppressing GIST-T1 cell proliferation. However, direct PDGFRA targeting and inhibition of migration and invasion were demonstrated specifically for miR-34a [62]. Moreover, miR-144-3p has been identified as part of predicted KIT/PLAT/ETV1-related circRNA–miRNA–mRNA regulatory networks in GISTs [77].

Additionally, miR-409-5p is downregulated in GIST and suppresses angiogenesis by directly targeting KDM4D and inhibiting HIF-1β/VEGF-A signaling. Its restoration enhanced the antiangiogenic effect of imatinib, without demonstrated reversal of established resistance [82]. Within this axis, miR-6838-5p acts as a tumor-suppressive mediator, as its inhibition derepresses CDV3 and promotes GIST cell proliferation and aerobic glycolysis [81]. Furthermore, miR-186-5p exhibits metastasis-suppressive activity in GIST. Low expression was associated with metastatic recurrence and poorer survival in a 100-patient cohort, while its inhibition promoted GIST-T1 cell migration without affecting proliferation [80]. Moreover, miR-133b was downregulated in high-grade GISTs and inversely associated with FSCN1 expression, although direct miR-133b–FSCN1 regulation was not functionally validated in GIST models [84]. Furthermore, let-7e-5p is downregulated in relapsed compared with non-relapsed high-risk intestinal GISTs, and its loss is associated with poorer relapse-free survival and derepression of differentiation- and extracellular matrix-related genes [78]. Additional evidence supports a tumor-suppressive role for miR-137-3p, which inhibits epithelial–mesenchymal transition (EMT) and cellular proliferation through direct targeting of Twist1 [79].

### 7.4. Oncogenic miRNAs in GISTs

In addition to tumor-suppressive miRNAs, several miRNAs function as oncogenic regulators in GISTs and contribute to tumor progression, metastasis, and adverse clinical outcomes (Table 2). For instance, miR-25-3p is markedly upregulated in GISTs, with its maturation enhanced by METTL3-mediated m6A modification. Functionally, miR-25-3p promotes proliferation, migration, and colony formation through upregulation of CDK2, Cyclin D1, MMP2, and MMP9 [63]. Moreover, miR-28-5p has been proposed as a candidate regulator of imatinib response, although functional validation in GIST models remains limited [83].

miR-196a and the lncRNA HOTAIR are frequently co-upregulated in high-risk GISTs, where their expression is associated with larger tumor size, increased mitotic activity, metastasis, and poor prognosis [85]. Mechanistically, miR-196a enhances invasiveness by repressing targets such as ANXA1 and HOXB8, whereas HOTAIR promotes invasion through chromatin remodeling and PRC2-mediated silencing of tumor suppressor genes [85]. Importantly, inhibition of either molecule reduces GIST cell invasiveness, highlighting their cooperative oncogenic function [85].

Additional oncogenic miRNAs include miR-182, which is significantly upregulated in GISTs and promotes proliferation, migration, and survival by targeting the tumor suppressor CYLD and activating NF-κB signaling, whereas elevated miR-182 levels correlate with adverse clinicopathological features [86]. Similarly, miR-374b is markedly upregulated in GIST tissues and directly suppresses PTEN, leading to activation of the PI3K/AKT pathway. Functional studies demonstrate that miR-374b enhances proliferation, migration, invasion, and cell cycle progression while inhibiting apoptosis, and its expression correlates with larger tumor size and advanced pathological stage [87]. Interestingly, miR-520a-3p exhibits tumor-suppressive activity in GISTs by inhibiting cell proliferation and restoring imatinib sensitivity [65].

**Table 2:** Oncogenic microRNAs associated with GIST progression and metastasis. * Evidence currently limited to in silico analyses and/or lacking functional validation. ↑ indicates an increase in the indicated biological phenomenon.

miRNA	Functional Role	Molecular Target(s)/Pathway	Biological Effect	Reference
miR-28-5p *	Oncogenic miRNA/Imatinib response regulator	Predicted signaling targets	Associated with treatment response	[83]
miR-25-3p	Oncogenic	Downstream effectors are CDK2, Cyclin D1, MMP2, MMP9	↑ proliferation, migration	[63]
miR-196a	Oncogenic	ANXA1, HOXB8	↑ invasion, metastasis	[85]
miR-455-3p *	Oncogenic	Not established; correlated with H19 expression	Upregulated in KIT/PDGFRA-mutant GISTs; functional role not validated	[57,88]
miR-182	Oncogenic	CYLD/NF-κB pathway	↑ proliferation, survival	[86]
miR-374b	Oncogenic	PTEN/PI3K-AKT	↑ proliferation, invasion	[87]

## 8. Oncogenic and Tumor-Suppressive Roles of lncRNAs in GISTs

lncRNAs play diverse regulatory roles in GIST pathogenesis, progression, and therapeutic resistance, often converging on oncogenic signaling pathways, epigenetic regulation, and therapeutic response mechanisms (Table 3). Most reported lncRNAs display oncogenic or resistance-associated effects, whereas comparatively few have been proposed to exert potential tumor-suppressive roles (Table 4).

**Table 3:** Oncogenic lncRNAs reported to promote GIST progression through signaling, epigenetic, or ceRNA-mediated mechanisms. ^#^ Low statistical support in the source study.

lncRNA	Mechanism	References
DNM3OS	Upregulated in high-risk GIST; affects Hippo signaling leading to poor survival	[89]
SNHG16	Upregulated; sponges miR-128-3p; promotes proliferation, invasion and inhibits apoptosis	[64]
HOTAIR	Upregulated in high-risk GISTs; regulates ECM genes, alters DNA methylation (e.g., *RASSF1*); Sponges miR-130a-3p, thereby upregulating *ATG2B* and inducing autophagy	[73,85,90]
H19 ^#^	Upregulated; correlates with *ETV1* and miR-455-3p	[6,57]
HIF1A-AS2	Upregulated; implicated in proliferation via hypoxia/HIF signaling; may promote autophagy and reduce apoptosis	[6,91]
PVT1	Upregulated; promotes proliferation, migration, and survival; activates the PI3K/AKT/mTOR pathway	[6,59]
XIST	Upregulated; FTO demethylation stabilizes XIST to sponge miR-520a-3p, protecting oncogenic KIT	[65]
MALAT1 ^#^	Upregulated; Putative role in invasion	[57]
RP11-616M22.7	Upregulated in resistant GISTs; interacts with *RASSF1*; Hippo signaling	[92]
OIP5-AS1	Upregulated; exosomal lncRNA enriched in sunitinib-resistant GISTs; sponges miR-145 and upregulates *SOX9*	[93]
SPRY4-IT1	Upregulated in high-risk GISTs; sponges miR-101-5p to derepress ZEB1/ERK signaling, promoting proliferation, and invasion	[94]
MSC-AS1	Upregulated in imatinib-resistant GISTs; sponges miR-200b-3p to derepress FNDC1 and ANLN, activating PI3K/AKT	[95]
PCAT6	Upregulated in GISTs; promotes proliferation, stemness, and survival; Sponges miR-143-3p, upregulates *PRDX5*, activates Wnt/β-catenin signaling	[96]
LINC00870	Upregulated in imatinib-resistant GISTs; Binds PIGR, inhibits glycosylation; promotes proliferation	[97]

**Table 4:** lncRNAs with potential tumor-suppressive roles in GISTs, proposed to restrain tumor growth and improve treatment response, with reported mechanisms and supporting clinical evidence where available.

lncRNA	Mechanism	References
CCDC26	Downregulation leads to increased *IGF-1R* expression	[27]
FENDRR	Altered expression in GIST tissues; functional role not fully defined	[57]
MEG3	Epigenetically deregulated in GISTs; precise functional effects remain incompletely established	[98]

### 8.1. lncRNAs Regulating Oncogenic Signaling Pathways

Among these regulatory mechanisms, Hippo signaling has emerged as a key axis modulated by lncRNAs. DNM3OS is overexpressed in high-risk GISTs and correlates with shorter progression-free survival (PFS) and OS, promoting proliferation and mitosis partly via Hippo signaling and metabolic regulators such as GLUT4 and CD36 [89]. In a non-GIST chondrocyte model, DNM3OS was linked to miR-126-5p sponging and IGF1 upregulation [99]. However, whether this mechanism applies to GIST remains unknown.

MALAT1 and H19 are upregulated in GIST tissues, with H19 expression strongly correlating with the oncogene ETV1 and the oncogenic miRNA miR-455-3p [57]. Although the mechanistic link between H19 and miR-455-3p remains unclear, miR-455-3p is upregulated in KIT/PDGFRA-mutant GISTs, but its functional role in GIST has not been validated [57]. LncRNA-mediated regulation of Hippo signaling is further illustrated by RP11-616M22.7, which is upregulated in imatinib-resistant GISTs and promotes proliferation, invasion, and resistance through interaction with RASSF1 [92].

### 8.2. lncRNA-Mediated ceRNA Networks and Intercellular Communication

Several lncRNAs contribute to GIST progression through ceRNA interactions. For example, exosomal OIP5-AS1 promotes aggressive tumor behavior by sponging miR-145 and upregulating SOX9, thereby promoting pro-survival signaling and intercellular propagation of oncogenic programs [93].

Similarly, SPRY4-IT1 is markedly upregulated in high-risk and metastatic GISTs, where it promotes proliferation, invasion, and EMT by sponging miR-101-5p, derepressing ZEB1, and activating ERK signaling [94]. Interestingly, this ceRNA activity counteracts the tumor-suppressive function of its host gene SPRY4 [94].

MSC-AS1 promotes GIST cell survival and invasion by sponging miR-200b-3p, thereby derepressing FNDC1 and ANLN and activating PI3K/AKT signaling [95]. Likewise, PCAT6 exerts oncogenic effects by sponging miR-143-3p, upregulating PRDX5, and activating Wnt/β-catenin signaling, thereby enhancing proliferation, stemness, and survival [96]. Furthermore, LINC00870 is co-upregulated with PIGR in GISTs and is associated with poor prognosis. Mechanistically, the LINC00870–PIGR axis promotes proliferation, invasion, and metastasis in GIST cells [97].

### 8.3. lncRNAs Regulating Hypoxia Responses and Survival Signaling

Additional lncRNAs further contribute to GIST biology. Under hypoxic conditions, HIF1A-AS2 supports GIST cell survival by promoting autophagy and inhibiting apoptosis [6,91]. Similarly, PVT1 functions as an oncogenic regulator by activating PI3K/AKT/mTOR signaling, thereby promoting proliferation and migration while suppressing apoptosis [6,59].

### 8.4. Epigenetic and Epitranscriptomic Regulation Involving lncRNAs

Epigenetic regulation by lncRNAs is exemplified by HOTAIR, which is upregulated in high-risk GISTs and correlates with tumor size, mitotic index, and anatomical site. HOTAIR promotes invasiveness through PRC2-dependent DNA methylation changes, including silencing of tumor suppressors such as RASSF1, ALDH1A3, and PCDH10 [90,100]. At the post-transcriptional level, XIST stability is enhanced by FTO-mediated m6A demethylation [65]. Stabilized XIST acts as a molecular sponge for the tumor-suppressive miR-520a-3p, thereby protecting KIT from repression and sustaining oncogenic signaling pathways required for GIST cell proliferation and survival [65].

### 8.5. LncRNAs with Potential Tumor-Suppressive Roles in GISTs

Several lncRNAs may have tumor-suppressive or incompletely characterized roles in GISTs (Table 4). CCDC26 has also been implicated in imatinib resistance in GISTs through IGF-1R upregulation [27]. Transcriptomic datasets have also identified FENDRR as upregulated in GIST tissues [57]. However, its functional role in GIST biology requires further clarification. Finally, MEG3 appears to be epigenetically deregulated in GISTs, although its precise functional contribution remains incompletely established [98].

## 9. circRNAs in GIST Progression and Drug Resistance

### 9.1. circRNAs Regulating Oncogenic Signaling and Tumor Progression

Recent studies have identified circRNAs as additional regulators of GIST progression and therapy response (Table 5). Although circ_0069765, circ_0079471, and circ_0084097 are upregulated in GIST and arise from the KIT, ETV1, and PLAT loci, respectively, their inclusion in a computationally predicted circRNA–miRNA–mRNA network does not establish a shared pro-oncogenic function. In contrast, circ_0084097 was inversely associated with metastasis, and PLAT expression was negatively correlated with tumor size. Because these observations are correlative and have not been functionally validated, the role of circ_0084097 in GIST remains unresolved and requires further investigation [77]. Assessing the importance of circRNAs, an array-based profiling study identified 543 candidate differentially expressed circRNAs and validated six of them in 20 paired GIST and adjacent tissues. hsa_circRNA_061346, hsa_circRNA_103114, and hsa_circRNA_103870, subsequently termed circ_SMA4, were upregulated, whereas hsa_circRNA_405324, hsa_circRNA_406821, and hsa_circRNA_000361 were downregulated. Their clinicopathological associations and predicted circRNA–miRNA–mRNA networks suggest potential biomarker relevance, although functional roles and direct molecular interactions were not established in this study [101].

More recently, the APP-derived circRNA circAPP was found to be upregulated in GIST tissues and to promote proliferation, migration, invasion, and aerobic glycolysis by sponging miR-6838-5p and derepressing CDV3. circAPP silencing increased apoptosis and suppressed tumor growth and liver metastasis in vivo, supporting a functionally validated oncogenic role [81].

### 9.2. circRNAs Associated with Therapeutic Resistance and Clinical Biomarkers

Clinically relevant circRNAs have also been identified in patient samples. circ-CCS, detected at elevated levels in the serum of recurrent GIST patients, may promote autophagy in GIST-882 and GIST-T1 cells by sponging miR-197-3p, thereby potentially influencing ATG10 expression [6,102]. Similarly, circ_SMA4 promotes proliferation, invasion, and migration by sequestering miR-494-3p, thereby increasing KIT and activating JAK/STAT. The oncogenic role is supported in vitro and in vivo and suggests potential diagnostic value [103]. More recently, circ-EPHB4 (circ_0081518), circ-BRIP1 (circ_0045015), and circ-RECQL4 (circ_0086139) were consistently upregulated in imatinib-resistant GISTs across validation and independent testing cohorts. All three were associated with imatinib resistance and tumor relapse or progression, while circ-EPHB4 and circ-BRIP1 were additionally associated with metastasis [104]. High circ-EPHB4 expression was also associated with shorter progression-free survival. However, functional experiments are necessary to establish a causal role or direct regulatory mechanism [104]. These findings highlight circRNAs as emerging regulators of both tumor progression and therapeutic resistance in GISTs through modulation of ceRNA networks and survival signaling pathways.

## 10. ncRNA-Mediated Mechanisms of Drug Resistance in GISTs

Accumulating evidence indicates that ncRNAs constitute a critical regulatory layer underlying primary and acquired resistance to tyrosine kinase inhibitors in GISTs, particularly imatinib and sunitinib. These molecules influence drug response through multiple mechanisms, including modulation of KIT signaling, autophagy, survival pathways, and intercellular communication (Table 6).

### 10.1. miRNAs Associated with TKI Resistance

Several miRNAs have been directly linked to imatinib resistance. Increased expression of miR-28-5p has been associated with imatinib resistance in patient-derived datasets, although functional validation in GIST models remains limited [83]. Similarly, miR-125a-5p has been proposed as a regulator of imatinib response based on bioinformatic analyses [83] and has also been reported to be upregulated in resistant GISTs, where it may promote resistance by repressing PTPN18, particularly in KIT-mutant tumors [70]. Furthermore, miR-518a-5p was found to be downregulated in imatinib-resistant GISTs, and its loss may promote resistance through derepression of its validated target PIK3C2A, whereas restoration of miR-518a-5p in GIST882R cells reduced proliferation and increased apoptosis [74]. Loss of miR-30a-5p promotes autophagy-dependent survival. Restoration of miR-30a-5p expression sensitizes GIST-T1 and GIST-882 cells to imatinib by targeting BECLIN-1 and suppressing autophagy in both in vitro and in vivo models [72]. Restoration of miR-409-5p also enhanced imatinib-mediated inhibition of angiogenesis through suppression of the KDM4D/HIF-1β/VEGF-A axis, although reversal of an established resistant phenotype was not demonstrated [82]. Additional miRNAs have also been linked to therapeutic response. Reduced let-7e-5p expression showed a non-significant trend toward shorter progression-free survival after imatinib initiation [78], while miR-320a-3p downregulation in patient cohorts correlates with a markedly shorter time to resistance [105]. Moreover, serum miR-518e-5p was elevated in patients with secondary imatinib-resistant GIST and distinguished resistant from imatinib-sensitive cases in a retrospective cohort, supporting its potential as a circulating resistance-associated biomarker. However, no functional role in resistance was demonstrated [106].

Imatinib treatment was also associated with reduced miR-483-3p expression in GIST tissues and cell lines. Functional modulation of miR-483-3p altered mitochondrial Complex II/SDHB protein expression, while its overexpression attenuated imatinib-induced cell death. These findings implicate miR-483-3p in treatment-associated OXPHOS remodeling, although the direct SDH target and its net contribution to acquired resistance remain unresolved [107].

### 10.2. lncRNAs Mediating Therapeutic Resistance

LncRNAs play a prominent role in mediating resistance mechanisms in GISTs. The oncogenic lncRNA HOTAIR, which is upregulated in recurrent GISTs, promotes imatinib resistance by sponging miR-130a-3p and enabling ATG2B-mediated autophagy activation [73]. This mechanism has been validated in GIST-T1 and GIST-882 cells, as well as in xenograft models [73].

Similarly, RP11-616M22.7 is upregulated in imatinib-resistant GISTs and appears to sustain resistance programs by interacting with RASSF1 and altering Hippo pathway signaling [92]. Other lncRNAs contribute to resistance through alternative mechanisms. CCDC26 downregulation increases IGF-1R expression and promotes resistance to imatinib [27]. HIF1A-AS2 enhances resistance under hypoxic conditions by promoting autophagy and suppressing apoptosis [6,91]. Likewise, MSC-AS1 is upregulated in imatinib-resistant GISTs and promotes resistance by acting as a ceRNA for miR-200b-3p, leading to derepression of FNDC1 and ANLN, and activation of PI3K/AKT signaling [95]. Silencing MSC-AS1 restores imatinib sensitivity [95].

LINC00870 also contributes to resistance and metastasis via interaction with PIGR, while inhibition of this axis restores drug sensitivity [97]. Resistance mechanisms may also involve intercellular communication, as demonstrated by the exosomal lncRNA OIP5-AS1, which is enriched in sunitinib-resistant GISTs and promotes resistance through the miR-145/SOX9 axis [93]. Additionally, the RNA demethylase FTO stabilizes the lncRNA XIST by reducing its m6A modification and preventing YTHDF2-mediated degradation. Stabilized XIST functions as a ceRNA that sponges miR-520a-3p, maintaining high KIT expression even in the presence of imatinib [65].

### 10.3. circRNAs Contributing to Drug Resistance

CircRNAs also contribute to therapeutic resistance in GISTs. circ-CCS is elevated in the serum of imatinib-resistant and recurrent patients and promotes resistance in GIST-882 and GIST-T1 cells by sponging miR-197-3p, which leads to ATG10 upregulation and increased autophagy [6,102]. Together, these findings highlight the complex regulatory networks through which ncRNAs modulate therapeutic response in GISTs and underscore their potential value as predictive biomarkers and therapeutic targets.

### 10.4. Ferroptosis-Related Mechanisms and Potential ncRNA Regulation

Ferroptosis is an iron-dependent form of regulated cell death driven by the accumulation of lipid peroxides. Its principal antioxidant defense is the system xc−–glutathione (GSH)–GPX4 axis, through which cystine uptake supports GSH synthesis and enables GPX4 to detoxify lipid hydroperoxides [108]. In GIST models, imatinib increases intracellular Fe^2+^ and lipid reactive oxygen species, reduces GSH levels, and promotes STUB1-mediated ubiquitination and degradation of GPX4 [109]. Combining imatinib with the GPX4 inhibitor RSL3 further enhances ferroptosis and suppresses GIST growth in vitro and in vivo [109], while GIST cells are also sensitive to ferroptosis induced by RSL3 and YAP-targeting agents that impair antioxidant defences [110].

ncRNAs can modulate ferroptotic susceptibility by regulating GPX4, SLC7A11, FTH1, ACSL4, and related iron- and lipid-metabolism pathways through direct miRNA targeting or lncRNA/circRNA-mediated interactions in many tumors [111]. However, these mechanisms have largely been established outside GIST, and no specific ncRNA–ferroptosis axis has yet been functionally validated in GIST models. Ferroptosis therefore represents a plausible but still unconfirmed link between ncRNA dysregulation and TKI response that requires dedicated mechanistic investigation.

## 11. Context-Dependent ncRNA Networks in GISTs

**Figure 3:**
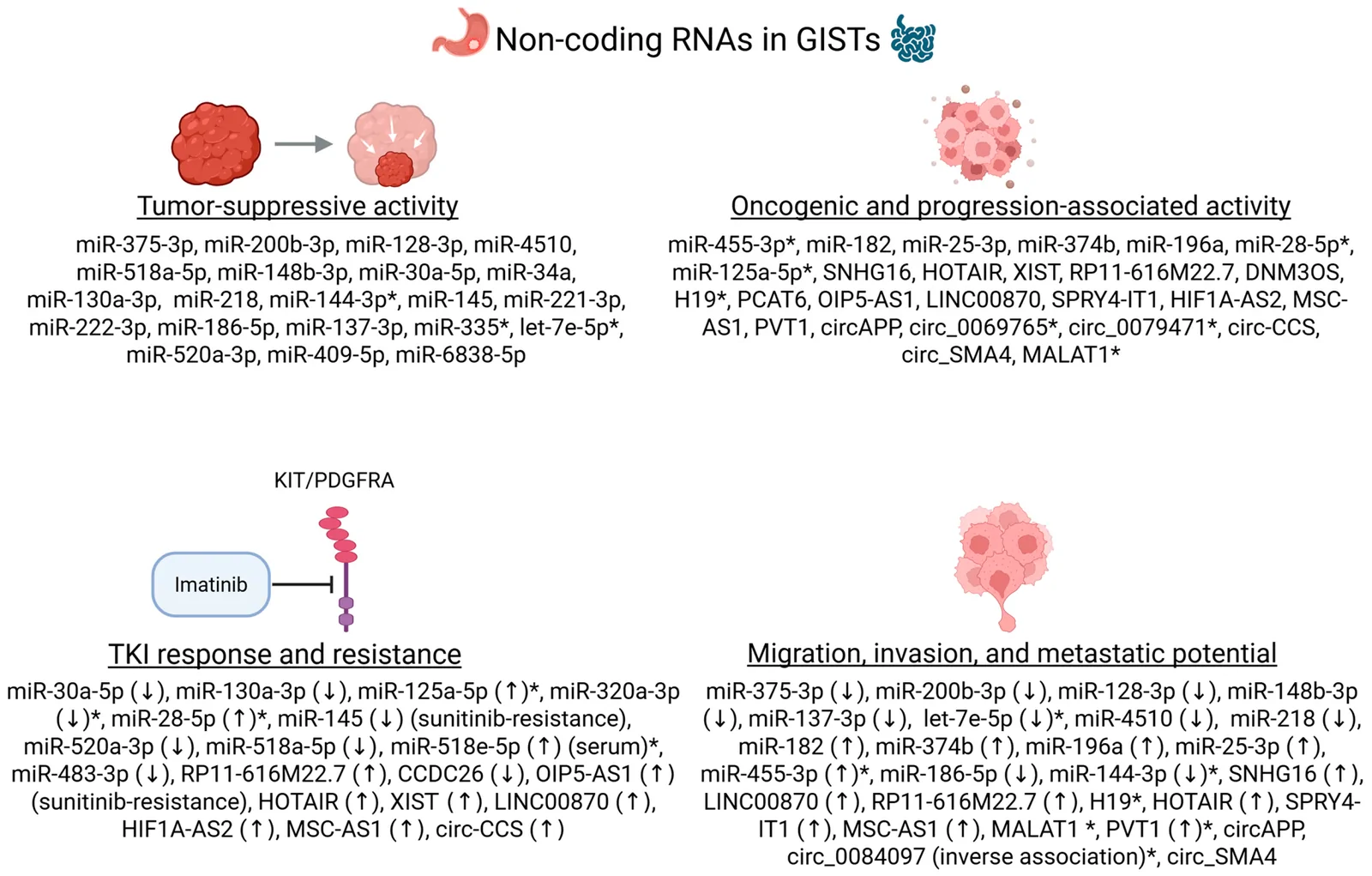
Schematic overview of ncRNA-mediated regulatory networks contributing to key biological processes in GISTs. Various miRNAs, lncRNAs, and circRNAs modulate tumor cell proliferation, invasion, survival, and response to TKIs through pathways including KIT/PI3K/AKT signaling, Hippo signaling, and ceRNA regulatory networks. Icons indicate the predominant anatomical origins of GISTs, including the stomach (**left**) and the small intestine (jejunum and ileum on the **right**). Putative, association-based, or primarily computationally predicted roles are indicated with an asterisk. For circ_0084097, the inverse association with metastasis is correlative, and its functional role remains unresolved. ↑ and ↓ indicate upregulated and downregulated expression, respectively, in relation to the indicated GIST phenotype. Created in BioRender. Mandrakis, G. (2026) https://BioRender.com/0v7sr2b (accessed on 12 August 2026).

The functional roles of ncRNAs in cancer are increasingly recognized as context-dependent, challenging the traditional classification of these molecules as strictly oncogenic or tumor-suppressive. In GISTs, ncRNA networks regulate key hallmarks of tumor biology, including proliferation, invasion, and therapeutic response, as summarized in Figure 3.

### 11.1. ncRNAs Regulating Invasion and Metastatic Potential

Several miRNAs have been associated with invasive and metastatic behavior in GISTs. Tumor-suppressive miRNAs such as miR-375-3p, miR-200b-3p, miR-128-3p, miR-148b-3p, miR-4510, and miR-218 reduce invasion when their expression is restored [64,67,71,75,76]. In contrast, oncogenic miRNAs including miR-25-3p, miR-182, miR-196a, and miR-374b promote or are associated with migration, invasion, and metastatic potential in GIST cells [63,85,86,87]. miR-455-3p is upregulated in KIT/PDGFRA-mutant GISTs, but its contribution to these phenotypes has not been functionally validated [57]. Loss of miR-186-5p also promoted GIST-T1 cell migration and was associated with metastatic recurrence in patient cohorts [80].

lncRNAs also contribute to invasive behavior through ceRNA-mediated regulatory networks. Pro-invasive lncRNAs in GISTs include HOTAIR, LINC00870, RP11-616M22.7, SNHG16, SPRY4-IT1, and MSC-AS1, which regulate pathways involved in epithelial–mesenchymal transition, proliferation, and survival [64,90,91,92,93,94,95,97,100]. circRNAs further expand this regulatory layer, showing that circ_0084097 is inversely associated with metastasis, and circ_SMA4 promotes proliferation, migration, invasion, and tumor progression, whereas circ-CCS is primarily linked to autophagy-mediated imatinib resistance [77,102,103].

### 11.2. Context-Dependent Roles in GIST Biology

The functional effects of ncRNAs in GISTs depend on molecular subtype, mutational background, and therapeutic setting. In GISTs, miR-125a-5p is best regarded as a context-dependent regulator of therapeutic response rather than a uniformly oncogenic or tumor-suppressive miRNA. Bioinformatic analyses have associated altered miR-125a-5p expression with imatinib response [83], whereas functional studies linked miR-125a-5p overexpression to PTPN18 repression and imatinib resistance, particularly in KIT-mutant GIST models [70].

A similar context dependency is observed for lncRNAs. H19 is upregulated and associated with oncogenic markers in GISTs, and HOTAIR, SNHG16, and SPRY4-IT1 have been functionally linked to aggressive tumor behavior [57,64,90,94,100], whereas resistance-associated lncRNAs including HIF1A-AS2, MSC-AS1, XIST, OIP5-AS1, and LINC00870 participate in tumor-specific regulatory circuits involving autophagy, PI3K/AKT signaling, KIT derepression, and exosomal communication [6,65,91,93,95,97]. Several circRNAs also appear to exert context-dependent effects that vary according to the dominant molecular network in GISTs [77,102,103]. Collectively, these observations indicate that ncRNAs converge on common oncogenic pathways in GISTs, while their functional effects remain strongly influenced by molecular and therapeutic context.

### 11.3. Clinical Translation of ncRNAs: Biomarkers, Therapeutic Targeting, and Current Challenges

Although serum miR-518e-5p and circ-CCS illustrate the potential of ncRNA-based liquid biopsy for detecting secondary imatinib resistance, neither has undergone prospective multicenter validation for routine clinical use [102,106]. Clinical implementation is hindered by heterogeneity in biospecimen collection and processing, RNA isolation, assay platforms, normalization strategies, and cut-off selection, which limits reproducibility and cross-study comparison [38]. Moreover, the available clinical evidence is derived mainly from small, retrospective, or single-center cohorts. Standardized pre-analytical and analytical workflows, prospectively defined thresholds, and independent validation in multicenter cohorts will therefore be required, together with demonstration of added value beyond established clinicopathological and molecular risk-stratification tools [19].

Therapeutic modulation of ncRNAs represents a potential strategy for complementing TKI treatment in GIST. Tumor-suppressive miRNAs could be restored using synthetic miRNA mimics, whereas oncogenic miRNAs could be inhibited using antagomirs or related antisense oligonucleotides. Similarly, oncogenic lncRNAs could be suppressed using antisense oligonucleotides (ASOs) or small interfering RNAs (siRNAs). In GIST models, restoration of miR-221 and miR-222 inhibits KIT/AKT signaling and induces apoptosis, while miR-30a restoration suppresses BECLIN-1-dependent autophagy and enhances imatinib sensitivity [68,72]. Experimental silencing of HOTAIR and MSC-AS1 likewise increases imatinib sensitivity, supporting these lncRNAs as potential therapeutic targets [73,95]. However, these findings remain preclinical proof of concept, and no ncRNA-directed therapy has yet been clinically validated in GIST.

**Figure 4:**
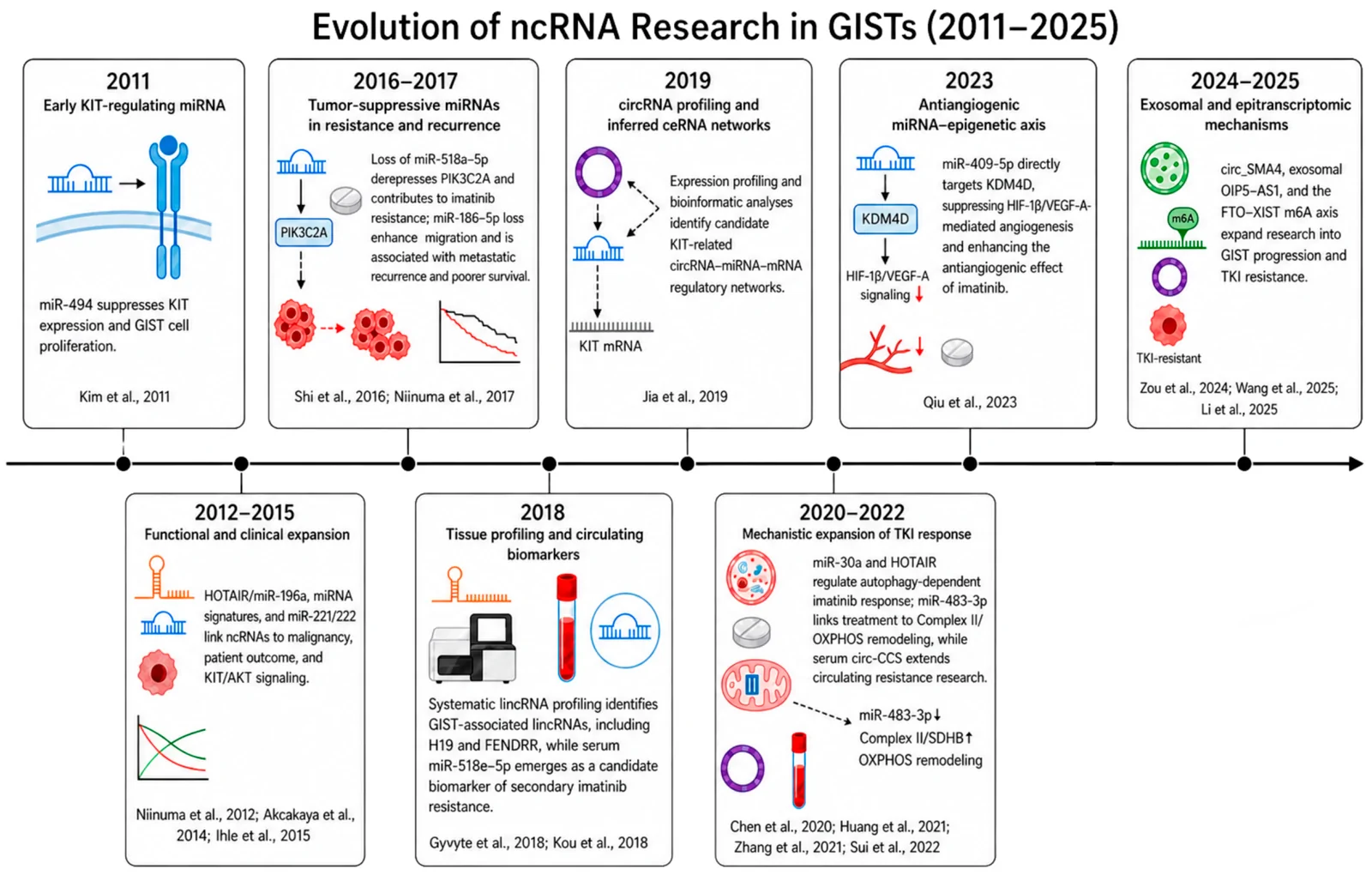
Representative milestones in the evolution of ncRNA research in GISTs from 2011 to 2025, encompassing early KIT-regulating miRNAs, clinical and transcriptomic profiling, inferred ceRNA networks, and reported mechanistic and translational findings related to tumor progression and TKI response. The timeline is illustrative rather than exhaustive and integrates evidence from patient material, cell-based models, and computational analyses. Schematic arrows do not imply a uniform level of causal validation. Circulating biomarker findings remain retrospective, whereas most TKI-response mechanisms are preclinical, and none of the depicted ncRNAs has undergone prospective multicenter validation. Created in BioRender. Mandrakis, G. (2026) https://BioRender.com/ef09254 (accessed on 12 August 2026). Kim et al. [69]; Niinuma et al. [85]; Akcakaya et al. [70]; Ihle et al. [68]; Shi et al. [74]; Niinuma et al. [80]; Gyvyte et al. [57]; Kou et al. [106]; Jia et al. [77]; Chen et al. [72]; Huang et al. [107]; Zhang et al. [73]; Sui et al. [102]; Qiu et al. [82]; Zou et al. [103]; Wang et al. [93]; and Li et al. [65].

The clinical translation of ncRNA-based therapeutics remains limited by RNA instability, insufficient tumor-selective delivery, poor cellular uptake and endosomal escape, off-target effects, and potential immune toxicity [112]. Early-phase oncology trials have nevertheless demonstrated the feasibility of systemic miRNA replacement. Liposomal MRX34 produced pharmacodynamic target modulation in patients with advanced solid tumors but was terminated following serious immune-mediated adverse events, whereas EGFR-targeted, miR-16-loaded minicells (TargomiRs) showed an acceptable safety profile and preliminary activity in malignant pleural mesothelioma [113,114]. Translation into GIST will therefore require optimized delivery systems, confirmation of target engagement in tumor tissue, and rigorous evaluation of ncRNA-directed agents in combination with TKIs. To provide a concise historical perspective, representative milestones in the evolution of ncRNA research in GIST are summarized in Figure 4.

## 12. Limitations of the Current Evidence

The available evidence on ncRNAs in GIST should be interpreted in light of several limitations. Functional investigations frequently rely on a small set of established cell lines—most commonly GIST-T1, GIST-882, and GIST-48—which may not fully represent the molecular diversity of KIT-mutant, PDGFRA-mutant, SDH-deficient, and other wild-type tumors [64,68,72,73]. Although some larger tissue series have been reported [89], many patient-based studies remain modest in size. Representative studies used three paired samples for initial profiling and 68 pairs for validation [77], 14 cases for discovery and 86 for validation [78], or 15 cases for sequencing and 22 for RT-qPCR validation [57]. Cross-study comparison is further complicated by heterogeneity in biospecimen type, clinicopathological annotation, RNA isolation and normalization procedures, expression platforms, and statistical thresholds, while many clinical studies remain retrospective and single-center. Furthermore, several reported associations between ncRNA expression and metastasis, relapse, survival, or TKI response remain observational [70,77,78], while the circRNA–miRNA–mRNA network derived from expression profiling was largely computationally constructed [77]. Consequently, standardized pre-analytical, analytical, and reporting procedures, validation in genetically diverse experimental models, and prospective evaluation in larger independent multicenter cohorts, together with direct mechanistic studies, are required before ncRNA biomarkers or therapeutic targets can be translated into clinical practice. To facilitate comparison across heterogeneous study designs, representative GIST-associated ncRNAs discussed in this review are summarized according to the type and depth of their supporting evidence and current translational status in Table 7.

## 13. Conclusions

ncRNAs have emerged as important regulators of GIST biology, capturing biologically meaningful heterogeneity that cannot be explained by KIT/PDGFRA mutational status alone. By modulating post-transcriptional regulation and ceRNA networks, ncRNAs influence clinically relevant processes including tumor invasion, metastasis, and resistance to TKIs. These regulatory mechanisms expand the biomarker landscape beyond protein-based readouts and provide new opportunities for molecular stratification.

Several ncRNAs have demonstrated clear functional relevance in GIST models. For example, miR-221-3p and miR-222-3p re-expression reduces KIT phosphorylation and downstream AKT/BCL2 signaling, promoting apoptosis, while miR-30a-5p restores imatinib sensitivity through inhibition of BECLIN-1-mediated autophagy. Among lncRNAs, HOTAIR promotes imatinib resistance through the miR-130a-3p/ATG2B autophagy axis, whereas MSC-AS1 and the LINC00870–PIGR signaling pathway have been linked to resistance mechanisms whose inhibition restores drug sensitivity. Additional molecules, including OIP5-AS1 and the circRNA circ-CCS, may serve as clinically accessible biomarkers, particularly given their detectability in patient-derived samples such as serum.

These observations support the concept that multiplex ncRNA profiling may offer greater clinical utility than single-marker approaches. A combined panel incorporating tumor-suppressive, oncogenic and resistance-associated ncRNAs, such as miR-221-3p, miR-222-3p, miR-518a-5p, miR-182, and miR-30a-5p, together with selected lncRNAs (HOTAIR, XIST, SNHG16, MSC-AS1, OIP5-AS1, LINC00870) and circRNAs such as circ-CCS, could potentially improve risk stratification and prediction of therapeutic response. Importantly, such panels are technically feasible using established RNA-based platforms, including RT-qPCR.

More broadly, the findings summarized in this review highlight the context-dependent nature of ncRNA regulatory networks in GISTs. Although different ncRNA–miRNA interactions may converge on shared oncogenic pathways, their specific functional consequences are strongly influenced by molecular subtype, therapeutic setting, and cellular context. Clinical translation nevertheless remains constrained by small and heterogeneous cohorts, variability in analytical workflows, and the lack of prospective multicenter validation. Future studies integrating ncRNA-focused assays with single-cell and spatial transcriptomics and complementary genomic, epigenomic, proteomic, and clinicopathological data may help resolve cell-type-specific regulatory programs and improve biomarker prioritization. Interpretable AI-assisted approaches may further support multimodal biomarker discovery, provided that models are developed using harmonized datasets and undergo rigorous external and prospective validation.

## Figures and Tables

**Table 5:** CircRNAs implicated in GIST progression and therapy response, reported in tissue and biofluids, emphasizing proposed ceRNA interactions and key downstream pathways.

circRNA	Proposed Mechanism/ceRNA Interaction	References
circ-CCS	Upregulated; sponges miR-197-3p and may influence autophagy by modulating ATG10	[6,102]
circ_0069765	Upregulated; derived from the KIT locus and participates in a KIT-centric ceRNA regulatory network	[77]
circ_0079471	Upregulated; derived from the ETV1 locus and engages ceRNA interactions with miRNAs	[77]
circ_0084097	Upregulated; derived from the PLAT locus and included in a computationally predicted ceRNA network; inversely associated with metastasis; functional role unresolved	[77]
circAPP	Upregulated; sponges miR-6838-5p to derepress CDV3, promoting proliferation, migration, invasion, the Warburg effect, tumor growth, and liver metastasis	[81]
circ_SMA4	Upregulated; Sponges miR-494-3p derepressing KIT and activates JAK/STAT pathway; promotes proliferation, migration, invasion, inhibits apoptosis	[103]

**Table 6:** ncRNAs linked to TKI resistance in GISTs, whose altered expression is associated with resistance or sensitization to TKIs. * Proposed target that requires further validation. ^#^ Evidence based mainly on in silico analyses.

ncRNA	Mechanism of Resistance/Effect on TKI Response	References
miR-28-5p ^#^	Overexpression is speculated to be associated with imatinib resistance	[83]
miR-320a-3p *	Downregulated expression is associated with earlier development of imatinib resistance	[105]
miR-125a-5p ^#^	Overexpression is associated with resistance in KIT-mutant tumors and resistant GISTs	[70,83]
miR-30a-5p	Downregulated expression supports autophagy-dependent survival and resistance, while upregulated expression improves response by suppressing autophagy	[72]
miR-34a *	Loss may promote PDGFRA-related signaling, but its effect on TKI response has not been directly evaluated in GISTs	[62]
miR-518a-5p	Downregulation derepresses PIK3C2A and contributes to imatinib resistance	[74]
miR-518e-5p *	Elevated in secondary imatinib-resistant GIST; candidate circulating biomarker without functional evidence of resistance mediation	[106]
miR-483-3p	Downregulated by imatinib; modulates Complex II/SDHB and OXPHOS remodeling, while overexpression attenuates imatinib-induced cell death; direct target unresolved	[107]
miR-409-5p	Restoration enhances the antiangiogenic effect of imatinib through the KDM4D/HIF1β/VEGF-A axis	[82]
let-7e-5p	Downregulation is associated with relapse and shows a non-significant trend toward shorter progression-free survival after imatinib initiation	[78]
CCDC26	Downregulated expression contributes to imatinib resistance through IGF-1R upregulation	[27]
HOTAIR	Upregulated expression promotes resistance through autophagy activation, supported by functional and xenograft evidence	[73]
XIST	Upregulated expression drives imatinib resistance by sponging miR-520a-3p derepressing KIT; silencing XIST drives imatinib sensitivity	[65]
RP11-616M22.7	Upregulated expression is associated with resistant GISTs and supports a resistant phenotype	[92]
OIP5-AS1	Upregulated expression is linked to sunitinib resistance via the miR-145/SOX9 axis, with exosomal enrichment suggesting a clinically accessible signal	[93]
MSC-AS1	Upregulated expression supports resistance, and silencing has been shown to restore sensitivity	[95]
LINC00870	Upregulated expression supports resistance through modulation of PIGR-associated signaling	[97]
HIF1A-AS2	Upregulated expression under hypoxia promotes resistance by enhancing autophagy and suppressing apoptosis; silencing improves sensitivity	[91]
circ-CCS	Upregulated expression promotes resistance through autophagy induction in cell models	[102]

**Table 7:** Evidence profiles of representative GIST-associated ncRNAs discussed in this review. ✓, evidence of the indicated type reported; D, evidence derived from public GIST datasets; —, not reported. “Cell lines” indicates that the ncRNA was examined in cultured GIST cells and may encompass expression measurements, phenotypic assays, or direct target interaction validation. Therefore, a check mark in this column does not necessarily indicate functional validation. For circ_0069765 and circ_0079471, cell-line evidence comprised expression assessment, whereas circ_0084097 was experimentally overexpressed in GIST-T1 cells. “Patient material” includes retrospective tissue, serum, or cohort-based findings and does not constitute prospective clinical validation. “Computational analysis” includes analyses of sequencing or omics data, pathway or enrichment analyses, and in silico target or interaction prediction. “Animal model” denotes in vivo functional assessment of the listed ncRNA and measurement as a downstream readout alone was not counted. None of the listed ncRNAs has yet undergone prospective multicenter validation as a GIST biomarker or therapeutic target.

ncRNA	Patient Material	Cell Lines	Animal Model	Computational Analysis	References
miR-455-3p	✓	—	—	✓	[57]
miR-34a	✓	✓	—	✓	[62]
miR-335	✓	✓	—	✓	[62]
miR-25-3p	✓	✓	—	—	[63]
miR-128-3p	—	✓	—	✓	[64]
miR-520a-3p	✓	✓	—	✓	[65]
miR-375-3p	—	✓	—	✓	[67]
miR-200b-3p	—	✓	—	✓	[67]
miR-221-3p	✓	✓	—	—	[68]
miR-222-3p	✓	✓	—	—	[68]
miR-494-3p	✓	✓	—	✓	[69]
miR-28-5p	—	—	—	✓ (D)	[83]
miR-125a-5p	—	—	—	✓ (D)	[83]
miR-125a-5p	✓	✓	—	✓	[70]
miR-148b-3p	✓	✓	✓	✓	[71]
miR-30a-5p	✓	✓	✓	✓	[72]
miR-130a-3p	—	✓	—	✓	[73]
miR-4510	✓	✓	—	✓	[75]
miR-218	✓	✓	—	—	[76]
miR-144-3p	—	—	—	✓	[77]
miR-133b	✓	—	—	—	[84]
miR-409-5p	✓	✓	—	✓	[82]
let-7e-5p	✓	—	—	—	[78]
miR-137-3p	✓	✓	—	✓	[79]
miR-196a	✓	✓	—	✓	[85]
miR-182	✓	✓	—	✓	[86]
miR-6838-5p	✓	✓	—	✓	[81]
miR-374b	✓	✓	—	✓	[87]
miR-145	✓	✓	✓	✓	[93]
miR-200b-3p	—	✓	—	✓	[95]
miR-101-5p	—	✓	—	✓	[94]
miR-143-3p	✓	✓	—	✓	[96]
miR-494-3p	✓	✓	—	✓	[103]
miR-320a-3p	✓	—	—	—	[105]
miR-186-5p	✓	✓	—	✓	[80]
miR-197-3p	—	✓	—	—	[102]
miR-518a-5p	✓	✓	—	✓	[74]
miR-518e-5p	✓	—	—	✓	[106]
miR-483-3p	✓	✓	—	✓	[107]
CCDC26	—	✓	—	—	[27]
SNHG16	✓	✓	✓	✓	[64]
XIST	✓	✓	✓	✓	[65]
H19	✓	—	—	✓	[57]
FENDRR	✓	—	—	✓	[57]
MALAT1	✓	—	—	✓	[57]
PVT1	—	✓	—	—	[59]
DNM3OS	✓	✓	—	✓	[89]
RP11-616M22.7	✓	✓	✓	✓	[92]
OIP5-AS1	✓	✓	✓	✓	[93]
SPRY4-IT1	✓	✓	—	✓	[94]
MSC-AS1	✓	✓	✓	✓	[95]
PCAT6	✓	✓	—	✓	[96]
LINC00870	✓	✓	✓	✓	[97]
HIF1A-AS2	✓	✓	—	—	[91]
HOTAIR	—	✓	✓	✓	[73]
HOTAIR	✓	✓	—	✓	[85]
HOTAIR	✓	✓	—	✓	[90]
HOTAIR	✓	✓	—	✓	[100]
MEG3	✓	✓	—	✓	[98]
circ_0069765	✓	✓	—	✓	[77]
circ_0079471	✓	✓	—	✓	[77]
circ_0084097	✓	✓	—	✓	[77]
circAPP	✓	✓	✓	✓	[81]
circ-CCS	✓	✓	—	—	[102]
circ_SMA4	✓	✓	✓	✓	[103]

## Data Availability

No new data were created or analyzed in this study. Data sharing is not applicable to this article.
